# Expression Changes of miRNAs in Humans and Animal Models of Amyotrophic Lateral Sclerosis and Their Potential Application for Clinical Diagnosis

**DOI:** 10.3390/life14091125

**Published:** 2024-09-06

**Authors:** Ruili Wang, Liang Chen, Yuning Zhang, Bo Sun, Mengyao Liang

**Affiliations:** College of Bioengineering, Beijing Polytechnic, Beijing 100176, China

**Keywords:** amyotrophic lateral sclerosis, miRNAs, biomarkers, animal models, detection

## Abstract

Amyotrophic lateral sclerosis (ALS) is a severe motor neuron disease. Current detection methods can only confirm the diagnosis at the onset of the disease, missing the critical window for early treatment. Recent studies using animal models have found that detecting changes in miRNA sites can predict the onset and severity of the disease in its early stages, facilitating early diagnosis and treatment. miRNAs show expression changes in motor neurons that connect the brain, spinal cord, and brain stem, as well as in the skeletal muscle in mouse models of ALS. Clinically, expression changes in some miRNAs in patients align with those in mouse models, such as the upregulation of miR-29b in the brain and the upregulation of miR-206 in the skeletal muscle. This study provides an overview of some miRNA study findings in humans as well as in animal models, including SOD1, FUS, TDP-43, and C9orf72 transgenic mice and wobbler mice, highlighting the potential of miRNAs as diagnostic markers for ALS. miR-21 and miR-206 are aberrantly expressed in both mouse model and patient samples, positioning them as key potential diagnostic markers in ALS. Additionally, miR-29a, miR-29b, miR-181a, and miR-142-3p have shown aberrant expression in both types of samples and show promise as clinical targets for ALS. Finally, miR-1197 and miR-486b-5p have been recently identified as aberrantly expressed miRNAs in mouse models for ALS, although further studies are needed to determine their viability as diagnostic targets.

## 1. Introduction

**Figure 1:**
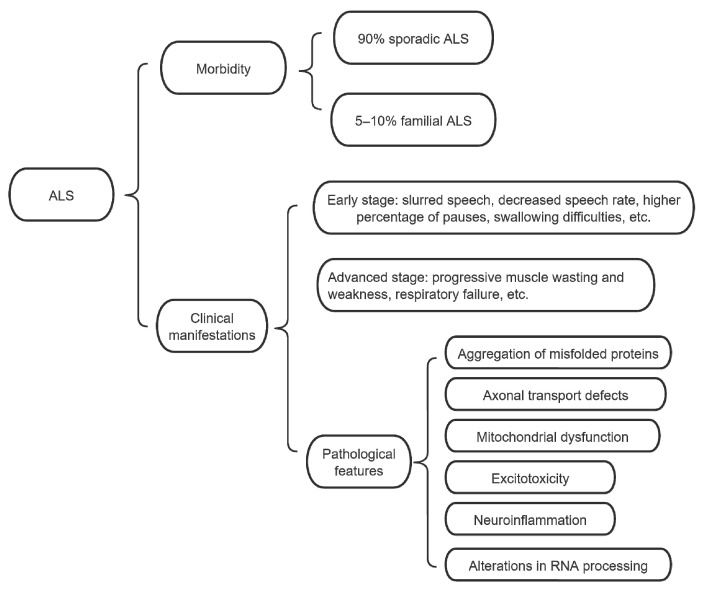
Morbidity and clinical manifestations of amyotrophic lateral sclerosis (ALS).

Amyotrophic lateral sclerosis (ALS), also known as Lou Gehrig’s disease, is the most common motor nerve disease in adults, with an annual incidence of 2–3 per 100,000 individuals worldwide. It manifests clinically as progressive myoatrophy and weakness. Bulbar symptoms are hallmarks of ALS. Early on, 25–30% of patients present with slurred speech, decreased speed of articulatory organ movement, decreased speech rate, and a higher percentage of pauses. Most patients (85%) experience swallowing difficulties. In addition to swallowing-related muscle weakness, decreased pharyngeal sensation and significantly impaired cough reflex may be the main causes of dysphagia in patients with ALS [1,2,3]. As the disease progresses, patients exhibit progressive muscle wasting and weakness, ultimately leading to death from respiratory failure. The main pathological characteristics include aggregation of misfolded proteins, axonal transport defects, mitochondrial dysfunction, excitotoxicity, neuroinflammation and alterations in RNA processing [1,3,4]. Most cases of ALS are sporadic, with only 5–10% of cases being familial ALS (fALS) [5,6,7], as shown in Figure 1.

Recent studies have shown that ALS is not only a movement disorder, but also features cognitive deficits and behavioral manifestations similar to those observed in frontotemporal dementia [8]. Its pathogenesis involves multiple systems, including the immune and nervous systems. In both familial and sporadic ALS, the onset of the disease is closely linked to the degeneration of functional proteins, and genetic mutations are the major cause of the loss of function of these proteins. Previous studies have identified more than 20 genes associated with the development of ALS, including Cu/Zn superoxide dismutase 1 (SOD1), TAR DNA binding protein-43 (TDP43), fused in sarcoma/translocated in liposarcoma (FUS), chromosome 9 open reading frame 72 (C9ORF72), and profilin 1 (PFN1). Mutations in these genes have been reported in approximately 60–70% of fALS (familial) and 10% of sALS (sporadic) cases [9]. The complexity of ALS pathogenesis poses great difficulties for clinical diagnosis and treatment.

ALS is characterized by significant heterogeneity, and patients often experience a long delay from onset to diagnosis. This creates barriers to early detection and timely treatment, delaying the optimal time for treatment. Currently, the diagnostic tools for ALS include multiple methods, such as electrophysiology [10], imaging [11], and neurobiochemical markers [12]. With the continuous expansion of biomarker research, the diagnostic sensitivity and specificity of ALS have been significantly improved, which has had a positive role in promoting early diagnosis and treatment, disease progression monitoring, efficacy evaluation, and drug discovery and development. Consequently, miRNAs have recently been proposed as biomarkers for ALS. These RNA fragments regulate mRNAs for protein expression, leading to the use of miRNAs as a means of detecting the signs of ALS relatively early. Although some studies have reported on miRNAs as biomarkers for ALS in clinical diagnosis, many lack results from mouse models and fail to identify specific and new miRNAs with the potential to be diagnostic markers [7,13]. Therefore, in this paper, we review miRNAs expression in motor neurons (MNs) derived from mouse models for ALS and from patient samples and induced pluripotent stem cells (iPSCs)-derived MNs up to August 2024. Those miRNAs showing more promise for clinical applications are identified, as well as those that may represent novel research targets.

### 1.1. Hypotheses of Pathogenesis

**Figure 2:**
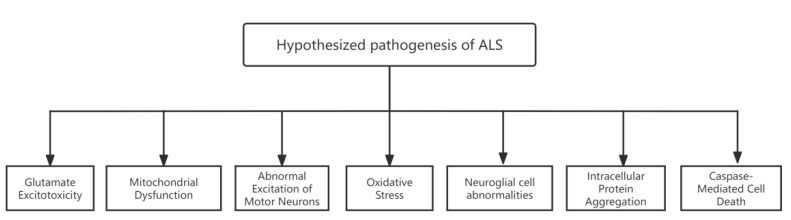
Hypothesized pathogenesis of amyotrophic lateral sclerosis (ALS).

The pathogenesis of ALS remains unclear; however, some hypotheses have been proposed, including glutamate excitotoxicity, where glutamate receptors are overexcited, leading to neuronal damage and death [14]. There are also morphologically and functionally abnormal mitochondria in the MNs of patients with ALS, and mitochondrial dysfunction may cause neuronal metabolic disorders resulting in cellular damage [15,16,17]. Increased excitability of MNs in mouse models for ALS may be associated with an abnormal increase in the sustained sodium current, a condition that exacerbates the process of oxidative stress damage and increases the mitochondrial metabolic load [18]. Mutations in genes or an aberrant expression of pathways associated with the oxidative stress response result in disruption of homeostasis and excessive accumulation of free radicals in the body, causing oxidative stress [19]. Additionally, in glial cell abnormalities [20], MNs are affected by astrocyte damage, and astrocytes with *SOD1* mutations secrete factors that are toxic to MNs, although the specific factors remain unidentified [21]. Intracellular protein aggregation, where the segregation of essential cellular components is disrupted in *Sod1* mice, may also be involved. Molecular chaperone activity is reduced in this model, and the ubiquitin-proteomic pathway, containing ubiquitin aggregates, exhibits decreased capacity [22]. Apoptosis is the mechanism of MNs death in *Sod1* mice. Caspase-1 and caspase-3 are activated sequentially in this mouse model of apoptosis [20] (Figure 2). In addition to the described hypotheses, the pathogenesis of ALS may be linked to immune-inflammatory responses [23], axial transport defects, and growth factors [24].

### 1.2. miRNAs as Potential Biomarkers for ALS

miRNAs are a type of non-coding RNA with a length of approximately 22 nucleotides. They are highly conserved and stable in animals, plants, and microorganisms. miRNAs are negative regulators of gene expression at the transcription level and are stable in the blood. They also possess highly tissue-specific properties, are quantifiable, and are easily extracted and analyzed [25,26]. Specifically, miRNAs have distinctive expression patterns in nerve cells; for example, miR-124 and miR-128 are expressed in neurons, while miR-23, miR-26, and miR-29 are expressed at higher levels in astrocytes than in neurons [26]. This suggests a correlation between miRNA expression and central nervous system diseases. miRNAs play a crucial role in neuroinflammation and are differentially expressed in ALS [27,28]. Previous studies have demonstrated that miRNAs have the potential as biomarkers for the diagnosis of neurological degenerative diseases. Recently, domestic and international studies have been conducted on miRNAs as diagnostic markers for neurological diseases. Some miRNAs have significance for the clinical diagnosis of ALS, assisting physicians in differential diagnosis and prognosis.

### 1.3. Roles of miRNAs in Pathogenesis of ALS

More than half of the protein-coding genes in an organism are regulated by miRNAs. Subsequently, miRNAs bind to specific regions of mRNAs and interfere with translation. A complete miRNA–mRNA match usually triggers degradation, while partial matches lead to translational repression [7]. Thus, miRNAs act as negative regulators of protein expression, with upregulated miRNAs leading to downregulation of target mRNAs in the corresponding samples, whereas downregulated miRNAs lead to upregulation of target mRNAs. A single miRNA can target hundreds of mRNAs, while a single mRNA can be targeted by multiple miRNAs [29].

Dash et al. showed that miRNAs targeting mRNAs are involved in biological processes, in molecular functions, and in the synthesis of cellular components. The target genes are mainly enriched in serine/threonine protein kinase complexes, salt/acid/ion/amine transport pathways, the p53 signaling pathway, and the homology box gene activation pathway [30].

Additionally, Vassileff et al. isolated miRNAs in serum and cerebrospinal fluid vesicles [31], while Rizzuti et al. isolated miRNAs in exosomes [32], demonstrating their crucial role in intercellular signaling. miRNAs regulate protein expression in ALS pathogenesis, and studying the changes in miRNA levels during disease progression may inform diagnosis and treatment strategies.

## 2. miRNA Research in Animal Models

### 2.1. Common Animal Models

ALS affects multiple systems, including the immune system, making animal models crucial to comprehensively studying the pathogenesis of ALS. Currently, there are two types of animal models used for ALS research: immune-induced mouse models and genetic models.

Mice treated with the plant sterol β-sitosterol-β-D-glucoside are commonly used as an inducible model [33]. However, these models are used less frequently compared to genetic models, possibly because the induced traits are less stable than those of genetic mouse models.

The wobbler mouse model, the first developed model for ALS, exhibits almost all clinical features of patients with ALS [34,35]. However, it is unsuitable for assessing frontotemporal dementia [36]. *SOD1* gene mutations or deletions are the most widely studied genetic causes of ALS [37], making *Sod1* transgenic mice the most widely used animal models. Consequently, mutations in *SOD1* can result in MN degeneration and myatrophy [38], leading to the current development of several *Sod1* mutant transgenic mouse models. The G93A mouse model, which overexpresses human mutant *SOD1*, exhibits neuropathological features and symptoms similar to those of patients with ALS [39,40]. The FUS mouse model overexpresses human wild-type *FUS* in the cell cytoplasm. Other FUS mouse models have also been developed, such as knockout mouse models lacking *Fus* [41]. The TDP-43-Q331K mouse model was established based on mildly overexpressing human *TDP43* mutants accompanied by a degenerative phenotype, whereas wild-type TDP-43 in humans does not cause this phenotype [42]. This mouse model exhibits symptoms similar to those of ALS, although it does not develop TDP-43 cytoplasmic aggregates or show TDP-43 nuclear export. Currently, more than 20 TDP-43 mouse models have been established [43]. Furthermore, the 72 open reading frame of human chromosome 9, whose mutation has been determined to be related to fALS, has 23–5000 hexanucleotide repeat expansions [44]. Consequently, many different rodent C9orf72 models have been developed, among which the FVB-C9orf72 BAC transgenic mice exhibit symptoms similar to human ALS [45]. The most common genetic causes of fALS are repeat amplification of the intronic region of *C9ORF72* and mutations in cytoplasmic SOD1. These factors account for approximately 70% of cases. [46,47]. Hence, *C9ORF72* mutations lead to the onset of fALS in approximately 40% of cases [48,49].

### 2.2. Research of miRNAs in Animal Models

We searched the PubMed database for relevant studies on ALS published before August 2024 using “ALS”, “mouse models”, and “miRNAs” as keywords, and performed a summarized analysis of the results. Table 1 shows the expression regions and trends of miRNAs in mouse models.

#### 2.2.1. Expression of miRNAs in the SOD1 Mouse Model

miR-29a: Nolan et al. [54] used RT-PCR to show that in the lumbar spinal cord of SOD1 (G93A) mice, miR-29a expression was increased before (approximately 70 d) and during onset (approximately 90 d) compared to *Sod1* wild-type mice. RT-PCR and in situ hybridization (ISH) results demonstrated that miR-29a was specifically expressed in the gray matter of the lumbar spinal cord. A recent study showed a correlation between miR-29a and cognitive performance in mice [69].

miR-29b family: Klatt et al. [56] found that miR-29b-3p expression in the spinal cord of wobbler mice was significantly reduced at birth and significantly upregulated at postnatal day 40 compared with that in wild-type mice. ISH analysis revealed that miR-29b-3p was mainly located in the spinal cord gray matter. The expression pattern of miR-29b-3p in cerebellar tissues showed similar dynamic changes. In wobbler mice, cerebellar miR-29b-3p expression was markedly decreased on postnatal day 0, but significantly increased by day 40. ISH results showed that miR-29b-3p-specific expression in mouse cerebellum was mainly concentrated in macrophages in the Purkinje cell layer. In addition, this study revealed that miR-29b-3p plays a key role in regulating the expression of Bcl-2 family members. This finding deepens our understanding of the functional complexity of the miR-29b family and provides valuable insight for future exploration of its specific mechanisms in apoptosis and related disease development. Yang et al. [70] used 16 SOD1 (G93A) mouse models and wild-type mice to detect miR-29b in the cerebral cortex, spinal cord, forelimb muscle tissue, and plasma by RT-PCR. The results demonstrated that the relative expression level of miR-29b in the cerebral cortex of the SOD1 (G93A) mouse model was markedly elevated in comparison to the control group. This finding is statistically significant and provides valuable insight to further investigate the mechanism of miR-29b in disease development [70].

**Figure 3:**
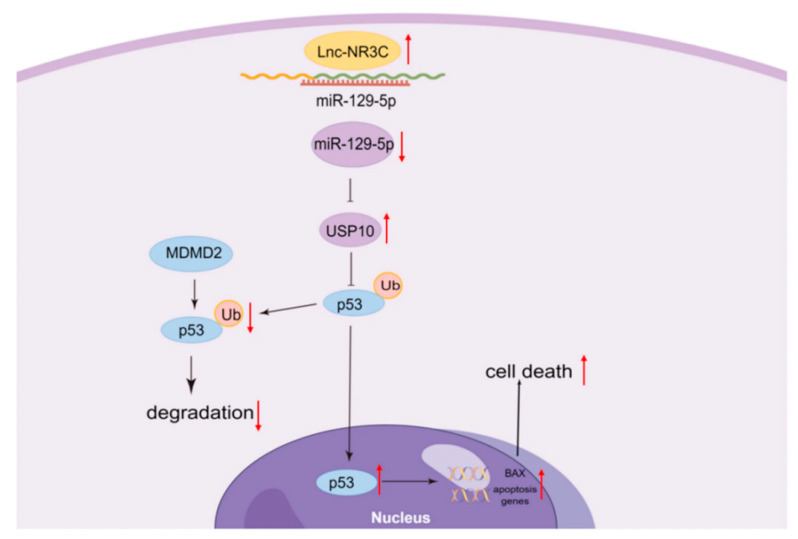
Model of miRNA-promoted apoptosis. The long non-coding RNA NR3C2-8:1(lnc-NR3C) promotes p53-mediated apoptosis through the miR-129-5p/USP10 Axis. Adapted from Ref. [60] with permission from Springer Link.

miR-200c andmiR-129-5p: Loffreda et al. [59] discovered by RT-PCR analysis that the expression of miR-200c and miR-129-5p was significantly upregulated in the spinal cord of SOD1 (G93A) transgenic female mice at early stages of the illness (15 weeks). This finding provides important clues for the early diagnosis of the illness and the development of potential therapeutic strategies. Pang et al. recently demonstrated that lnc-NR3C and miR-129-5 synergistically regulate USP10/p53 expression, thereby activating the p53 signaling pathway and ultimately triggering the phenomenon of apoptosis [60], as shown in Figure 3. Additionally, Zhang et al. showed that miR-200c achieves precise regulation of gene silencing through its specific targeting of ZEB1 [65].

miR-181a: Chen et al. [63] selected wild-type and SOD1 (G93A) transgenic mice at 95, 108, and 122 d, and examined expression changes in miR-181a and the miR-181a target gene *Caprin1* in the mouse spinal cord. The results showed that, compared to wild-type mice, miR-181a expression was increased in the spinal cord of ALS transgenic mice at 95, 108, and 122 d, and changes in *Caprin1* mRNA were not statistically significant. Studies by Ghorbani et al. demonstrated the regulatory role of miR-181a and miR-181b in CD4^+^ T cells, which effectively inhibited the process of Th1 generation. Additionally, miR-181a showed a promoting effect on Treg cell differentiation. Notably, both miR-181a and miR-181b targeted Smad7, enhancing our understanding of the functional mechanisms of these RNA molecules [64].

miRNA-132: Sun et al. [61] used spinal cord tissues of SOD1 (G93A) transgenic mice at the early (95 d), intermediate (108 d), and late (122 d) stages of illness and detected miRNA-132 using qRT-PCR and ISH. Compared with wild-type mice, the expression of miRNA-132 was lower in the spinal cord tissues of ALS mice. Positive miRNA-132 signals were mainly located in the cell bodies of anterior horn cells of the spinal cord. Using qRT-PCR and Western blotting techniques, changes in the mRNA and protein levels for the brain-derived neurotrophic factor (BDNF) were observed. Compared to wild-type mice, ALS transgenic mice had both BDNF mRNA and protein levels increased in their spinal cord tissues, and BDNF-immunopositive cells were mainly expressed in the anterior horn neurons, with significantly enhanced expression signals.

miR-124: Zhou et al. [57] studied the expression of miR-124 and its target genes *Sox2* and *Sox9*. qRT-PCR results showed that miR-124 was downregulated in the brainstem and spinal cord of SOD1 (G93A) ALS transgenic mice compared to wild-type mice. Additionally, miR-124 was downregulated in neural stem cells but upregulated in differentiated neural stem cells. Meanwhile, the protein levels of *Sox2* and *Sox9* showed opposite trends to those of the miR-124 levels, both in vitro and in vivo. Marcuzzo et al. [50] detected a marked increase in the expression of miR-124a in the whole brain of SOD1 (G93A) mice compared to wild-type *Sod1* control mice using RT-PCR. In SOD1 (G93A) primary motor cortex and brainstem motor cell nuclei, miR-124a expression was also markedly upregulated. Notably, the results of the two studies showed opposite results for miR-124a expression in the brainstem, highlighting the complexity and challenges of research in this area.

miR-17–92 family: Tung et al. [46] found that in the lumbar spinal cord of SOD1 (G93A) mice at 100 d, the expression of some members of the miR17–92 cluster (miR-18a, miR-17, miR-20a) was significantly reduced in MNs, and the expression of their targets (*Nedd4*-2, *Pten*, and *Ndfip1*) was significantly increased. This finding provides a new perspective for understanding the role of the miR-17–92 family in ALS, as well as a valuable reference for subsequent in-depth studies and the development of therapeutic strategies.

miR-155: The expression of miR-155 increased in the pre-symptomatic stage by RT-PCR, whereas the expression of cytokine signaling pathway inhibitor 1, its direct target, was decreased. miR-155 is markedly increased in the spinal cord of asymptomatic SOD1 (G93A) mice, indicating that the upregulation of miR-155 occurs before the onset of the disease [48,71]. In contrast, the expression of miR-155 in the cerebral cortex showed a significant downward trend during the onset period [72].

miR-218: Hoye et al. [62] confirmed the elevation of miR-218 in the spinal cord neurons of adult mice using ISH. In SOD1 (G93A) mice, disease onset typically occurs at 130 d, when the body weight reaches its peak. In miRNA-rich neurons in the spinal cord of SOD1 (G93A) mice, miR-218 and miR-138 began to decay daily at 126 d and reached a maximum in terminal decay. In the mouse models, surviving neurons showed no significant changes in miR-218 levels from pre-onset (70 d) to onset (140 d). The results suggest that miR-218 may be associated with MN injury in mice.

miR-125b: Parisi et al. [58] found that the expression of miR-125b in the lumbar spinal cord of SOD1 (G93A) mice at the terminal stage (approximately 23 weeks) showed a significantly increasing trend, similar to the expression of NADPH oxidase 2 mRNA, compared to that in non-transgenic mice. However, Western blotting analysis revealed a significant decrease in A20 protein levels in ALS mice compared to controls.

miR-9: Zhou et al. [51] found that in SOD1 (G93A) mice, miR-9 levels in the spinal cord were markedly higher than those in controls in the early (95 d), middle (108 d), and late (122 d) stages of the disease. ISH showed that miR-9 is located in the cytoplasm of gray matter cells in the spinal cord. Compared with wild-type mice, the number of miR-9-positive cells in SOD1 (G93A) mice increased significantly on days 95, 108, and 122. miR-9 is related to the differentiation and proliferation of neural stem cells. When investigating the function of miR-9 in SOD1 (G93A) mice, it is crucial to examine its specific effects on neurodegenerative lesions during disease progression. Given miR-9′s role in neural stem cell proliferation and differentiation, its aberrant expression may accelerate or slow pathological changes in neurodegenerative diseases by regulating these key cellular processes [51]. Using RT-PCR, Marcuzzo et al. observed that miR-124a expression was significantly increased in the whole brain of the SOD1 (G93A) model compared to brain samples from wild-type *Sod1* controls. Additionally, the expression of miR-124a was significantly upregulated in the brainstem motor nuclei and primary motor cortex regions of the SOD1 (G93A) model [50]. Chen et al. suggested that potential target genes of miR-9 may inhibit blood–spinal cord barrier disruption, neuroinflammation, and apoptosis in rat models through Map2k3- or Notch2-mediated signaling pathways [52].

miR-19: miR-19a and miR-19b expression was markedly increased in the whole brain of SOD1 (G93A) mutants compared to non-mutant *Sod1* control brain tissue, as detected by RT-PCR [50]. Han et al. demonstrated that miR-19 plays a regulatory role in adult neuronal development and regulates cell migration by directly targeting *Rapgef2* to regulate cell migration [24].

miR-146a and miR-21: In the cortical tissue of SOD1 (G93A) mice, the expression of miR-146a and miR-21was significantly reduced during the disease onset. miR-146a expression is also markedly reduced in the pre-disease stage, indicating that miR-146a may be an early biomarker for ALS [72,73]. 

miR-206: Williams et al. [71] discovered that miR-206 is a key regulatory factor for signal transduction in skeletal muscles and neurons and is a specific miRNA in skeletal muscles. During the development of miR-206-deficient mice, normal neurites can form in skeletal muscles, but miR-206 deficiency accelerates disease progression in SOD1 (G93A) ALS mice. miR-206 is necessary for synaptic regeneration after acute muscle nerve injury, which may explain its positive role in ALS. miR-206 promotes the compensatory regeneration of neuromuscular synapses through the regulation of fibroblast growth factor signaling pathways and histone acetylase 4 to slow the progression of ALS [66].

**Figure 4:**
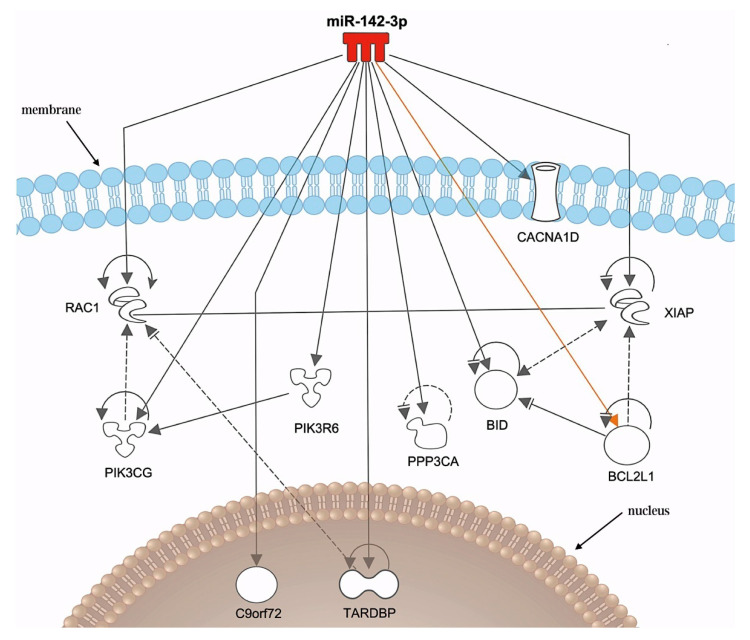
Molecular gene networks of ALS-related genes possibly modulated by miR-142-3p [48]. The 10 genes associated with ALS are presented as a gene network. The red color represents miRNA overexpression, which in this case is miR-142-3p. Black lines indicate miRNA/mRNA interactions. The orange line represents the regulation of a target gene, which has been validated by other research groups. The gray lines (solid and dashed) represent structural or functional relationships between genes or their encoded proteins. ALS, amyotrophic lateral sclerosis. Adapted from Ref. [47] with permission from Elsevier.

miR-142-3p: Matamala et al. [47] collected serum from *Sod1* transgenic mice, extracted RNA, and performed next-generation sequencing, which revealed significant changes in the expression of 471 miRNAs in SOD1 (G86R) mice and 405 miRNAs in SOD1 (G93A) mice compared to wild-type mice. In the late asymptomatic stage, miR-204-5p and miR-582-3p were co-expressed in two ALS experimental models, showing upregulation in SOD1 (G86R) mice and downregulation in SOD1 (G93A) mice. In the asymptomatic stage 2, miR-144-3p and miR-205-5p were co-expressed in the two ALS experimental models. miR-205-5p expression was downregulated in SOD1 (G86R) mice and upregulated in SOD1 (G93A) mice, whereas miR-144-3p expression was increased in both mouse models. The results showed that the miRNA expression profiles differed in mice with different mutations in the same gene. Highly expressed and conserved miRNAs were selected for qRT-PCR. Three of the seven miRNAs in SOD1 (G86R) showed significant changes in expression: miR-142-3p expression was increased while miR-205-5p and miR-375-3p expression were downregulated. Three of the six miRNAs verified in SOD1 (G93A) mice showed significant changes in expression, with miR-1249-3p and miR-183-5p expression upregulated and miR-204-5p expression downregulated. The six miRNAs were validated in TDP-43 (A315T) mice, and only miR-204-5p expression was significantly altered; that is, expression was downregulated. This differential expression also demonstrates the complexity of ALS pathogenesis. Cheng et al. [67] showed that miR-205-5p acts by directly targeting two transcription factors (ZEB1 and ZEB2). Matamala et al. [47] also found that miR-1249-3p and miR-142-3p are aberrantly expressed in ALS mice and the serum of ALS patients. Prediction of target genes of miR-1249-3p showed that they were related to axonal growth and the ephrin-B signaling pathway. Given the promise of miR-142-3p as a target, the authors analyzed its potential targets using IPA software (Ingenuity Systems, Redwood City, CA, USA). The results showed that miR-142-3p was associated with (i) genes related to mitochondrial dysfunction, apoptosis, and endoplasmic reticulum stress (*BCL2L1*, *BCL2*, *PIK3CG*, *PIK3R6*, and *CASP12*); (ii) genes associated with glutamate excitotoxicity (*CACNA1D*); and (iii) ALS-related genes such as *TDP43* and *C9orf72*. These results are summarized in Figure 4, demonstrating that miR-142-3p is closely associated with the pathogenesis of ALS. As shown in Figure 4, *CACNA1D* is anchored to the cell membrane; *BCL2L1*, *BCL2*, *PIK3CG*, *PIK3R6*, and *CASP12* are present in the cytoplasm; and *TDP-43* and *C9ORF72* are present in the nucleus.

#### 2.2.2. Expression of miRNAs in the Fus Mouse Model

*Fus* transgenic mice simulated the endogenous expression levels and patterns within the central nervous system (CNS). Ho et al. [55] established a miRNA library in the spinal cord and hippocampus of ALS-FUS mice using miRNA-seq, and determined that overexpression of *Fus* can cause different responses in mouse spinal cord and hippocampal neurons. Subsequently, they selected the following miRNAs for qRT-PCR validation: (i) miRNAs upregulated in the spinal cord and hippocampus, miR-119-3p, miR-323-3p, and miR-488-3p; (ii) miRNAs specifically upregulated in the spinal cord, miR-21a-5p and miR-203-3p; (iii) miRNAs specifically upregulated in the hippocampus, miR-488-5p; (iv) miRNAs specifically downregulated in the spinal cord, miR-484; and (v) miRNAs upregulated in the spinal cord and downregulated in the hippocampus, miR-146-5p. The qRT-PCR validation results demonstrate that miRNA-seq is not only consistent with the observed changes in qRT-PCR data but also comparable quantitatively. This proved that miRNA spectra can be used to study the molecular mechanisms of neurodegenerative diseases. Additionally, this team predicted and identified targets for miRNAs with abnormal expression: miR-1197-3p targeting Trib2, miR-496-3p targeting Mbnl1 and Cyp26b1, miR-488-3p targeting Vapb, miR-323-3p targeting Klf11 and Ubap2, and miR-410-3p targeting Slc25a12. Overexpression of miR-1197 reduces the level of Trib2 protein, confirming that upregulation and downregulation of miR-1197 decreases the expression of Trib2 in the spinal cord of ALS-FUS mice, although no change is observed in the hippocampus. Regulating Trib2 levels may provide additional therapeutic interventions for ALS.

#### 2.2.3. Expression of miRNAs in the TDP-43 (Q331K) Mouse Model

TDP-43 (Q331K) transgenic mice were generated by knocking down the mutant *Tdp43* (Q331K) gene.

miR-122-5p and miR-486b-5p: Vassileff et al. [31] isolated extracellular vesicles (evs) from the cortex and serum of 3- and 6-month-old TDP-43 (Q331K) and TDP-43 (WT) mice and extracted miRNAs. After isolating miRNAs, next-generation sequencing was performed. Compared with wild-type mice, abnormal expression of miR-122-5p and miR-486a-5p was detected in both cortical and serum tissues in TDP-43 (Q331K) model mice. miR-122-5p was downregulated in both tissues, while miR-486b-5p was upregulated in both tissues. Additionally, miR-183-5p was upregulated in serum. 

#### 2.2.4. Expression of miRNAs in the C9orf Mouse Model

C9orf transgenic mice are an ALS model created by cloning human *C9ORF* into BAC mice, which are viable semizygotic mice. miR-155 reduces the expression of C9BAC and polydipeptides in the C9orf mouse model. Peters et al. infected C9BAC mouse embryos with adenovirus containing miR-155. Compared with the control group, the expression of C9orf72 mRNA was reduced in all experimental groups, and the transcription of C9orf72 mRNA in a single embryo culture was reduced by 35% [49]. No other miRNAs have been reported in this mouse model.

## 3. Application Potential of miRNAs for Clinical Diagnosis

### 3.1. Expression of miRNAs in iPSC-Derived MNs of ALS Patients

Thus, the changes in miRNA expression in ALS mouse models have a pivotal role in the study of ALS pathogenesis. Alterations in miRNAs expression often precede changes in protein expression levels, suggesting that miRNAs could be used as early diagnostic markers for ALS. This will have a positive effect on ALS diagnosis, the monitoring of disease progression and treatment efficacy, and the development of novel therapeutic drugs. Rizzuti et al. [32] studied the miRNA expression profiles of iPSC-derived MNs from fALS patients and showed that miR-34a, miR-335, and miR-625-3p were deregulated in C9orf72-, SOD1-, and TARDBP-mutant MNs and in their exosomes. These miRNAs have been implicated in the regulation of several genes associated with apoptosis, synaptic plasticity, and mitochondrial generation. In addition, the authors analyzed the difference in expression of these miRNAs in the cerebrospinal fluid of patients and healthy individuals. miR-34a-3p and miR-625-3p expression was significantly upregulated in the cerebrospinal fluid of ALS patients compared to healthy individuals. These differential expressions offer the possibility of using miRNAs for clinical diagnosis. miR-218 was detected not only in the medium of MNs from ALS mouse models but also in the medium of MNs derived from iPSCs from fALS patients [62]. Dash et al. [30] analyzed transcriptomic and miRNA data from the same iPSC-derived MN samples from patients with ALS, mutant *SOD1*, TDP-43 proteins, and from healthy controls. Results indicated aberrantly regulated miRNAs such as miR-124-3p, miR-19b-3p, and miR-218, in MNs from patients with *SOD1* mutation. The differential expression of these miRNAs was closer to the in vivo expression reported in patients compared to those from the mouse model. miR-124-3p, miR-19b-3p, miR-218, and miR-335 were aberrantly expressed in both the *Sod1*-mutant mouse model and the iPSC-derived MNs of *SOD1*-mutant patients.

### 3.2. Expression of miRNAs in ALS Patients

Researchers have also investigated miRNA expression in human patients with ALS, revealing consistency between human and mouse models. In a study on the expression spectrum of miRNAs in the neurons of patients with Alzheimer’s disease and ALS, Shioya et al. [74] discovered that the expression of miR-29a, miR-29b, and miR-338-3p was upregulated in the frontal cortex of patients with ALS. This is consistent with the miR-29b upregulation in the brains of SOD1 (G93A) mice. In a study on the lumbar ventral cord of patients with ALS, the expression of miR-b1336 and miR-b2403 was downregulated, suggesting that miR-b1336 and miR-b2403 are associated with the onset of ALS [75]. Campos-Melo et al. [76] studied the expression spectrum of miRNAs in the lumbar ventral cord of patients with ALS and control groups and found that miR-558, miR16-2*, miR-146a*, and miR-508-5p were expressed in the spinal cord tissue of patients with ALS. Upregulation of miR-146a expression has also been detected in the mouse brain. In ALS onset accompanied by myoatrophy, miR-206 expression was found to be upregulated in a study of the expression spectrum of miRNAs in the skeletal muscle of patients [77,78,79]. In the SOD1 (G93A) mouse model, miR-206 in the skeletal muscle accelerates ALS progression. Additionally, miR-23a, miR-29b, and miR-455 were upregulated in skeletal muscle.

In the analysis of blood samples from patients, the expression of plasma RNA was found to be significantly increased in patients with ALS compared to healthy controls. Using a chip technique to analyze the expression spectrum of miRNAs, RT-qPCR revealed that miR-4649-5p expression was upregulated and miR-4299 expression was downregulated [80]. The expression of miR-1234-3p and miR-1825 is downregulated in the serum of carriers of fALS mutations [81]. In the cerebrospinal fluid, the expression of miR-181a-5p is upregulated, while that of miR-15b-5p, miR-21-5p, and miR-181a-5p is associated with ALS progression [82]. In a study on miR-338-3p, its expression was upregulated in the leukocytes, serum, cerebrospinal fluid, and spinal cord of patients with ALS [83]. Matamala et al. [47] showed that miR-142-3p expression was upregulated and miR-1249-3p expression was downregulated in ALS patients compared to controls, and both miR-142-3p and miR-1249-3p were aberrantly expressed in the SOD1 (G93A) mouse model.

By comparing serum samples from patients with ALS and healthy individuals, Joilin et al. [68] demonstrated significant changes in the expression of miR-16-5p, miR-21-5p, miR-92a-3p, and piR-33151 using qRT-PCR. Consequently, miR-206 showed a dichotomous expression pattern.

The findings suggest that these miRNAs are specifically expressed in different tissues of patients with ALS and can be used as clinical diagnostic markers, though their sensitivity and specificity require further verification. Consistent trends in expression levels of several miRNAs in mouse models and in human patients highlight the crucial role of mouse models in exploring the miRNA–ALS relationship and their diagnostic applications. Additionally, the miRNAs are expressed during the asymptomatic period of ALS, offering the potential for earlier disease detection compared to proteins and other biochemical markers. The use of miRNAs as diagnostic markers has important implications for the early detection, diagnosis, and treatment of diseases, as well as the prognosis of ALS.

### 3.3. Prediction of miRNAs by Bioinformatic Technology

Wang et al. [53] identified five miRNAs that could be used as biomarkers through bioinformatics (miR-221-5p, miR-21-5p, miR-100-5p, miR-30b-5p, and miR-615-3p). This study also utilized bioinformatics to explore the potential link between ALS and depression and identified potential biomarkers. Cheng et al. [67] proposed diagnostic rules by training the machine with miRNA keywords, including miR-205-5p, miR-206, and miR-376a-5p, while the key targets were BCL2, NEFH, and OPTN. The application of machine learning to miRNA-based diagnosis is also an important innovation of this study.

In summary, the complexity of ALS pathogenesis complicates accurate diagnosis, necessitating further studies and data to fully understand the disease. This article reviewed current studies on mouse models, iPSC-derived MNs, samples collected from patients, and bioinformatic predictions. miRNAs hold promise as clinical biomarkers. Abnormal expression of miR-21 and miR-206 has been consistently reported in both mouse models and patient samples, making them key potential diagnostic markers. miR-29a, miR-29b, miR-181a, and miR-142-3p have also been detected in mouse models and patient samples and are promising targets for clinical diagnosis. miR-1197 and miR-486b-5p are newly discovered miRNAs that are abnormally expressed in mouse models of ALS. As more targets and pathogenic mechanisms associated with ALS are reported, further verification on the sensitivity and specificity of miRNA expression is needed to access whether it can be used as a diagnostic marker. A considerable number of miRNAs have shown consistent trends in expression levels in mouse models and patients, implying crucial roles in the study of the relationship between miRNAs and ALS and the application of miRNAs in diagnosis. Additionally, miRNAs are expressed during the asymptomatic stage of ALS, which enables earlier detection and treatment of the disease compared to other advanced markers.

## 4. Conclusions

This article reviews the development of miRNAs as biomarkers for ALS, covering expression changes in iPSC-derived neurons and serum samples. The use of bioinformatic methods to predict miRNAs is also mentioned. The expression changes in miRNAs in early-stage ALS show potential for early detection and therapeutic intervention, but their sensitivity and specificity need further validation. More data are needed to support a deeper understanding of the role of miRNAs in the disease process. Animal models of ALS play a key role in research, and abnormal expression patterns of miRNAs provide important clues for clinical research. The potential of miRNAs as diagnostic markers and therapeutic targets is beginning to emerge. Research in animal models suggests that effective treatments for ALS are under active development. However, further studies and accumulation of data are needed to better understand the role of miRNAs in ALS pathogenesis.

## Figures and Tables

**Table 1:** Expression of miRNAs and target genes in animal models.

miRNA	Animal Model	Expression Region	Expression Result (↑ Indicates Upregulation)	Target Gene	Refs.
miR-9	SOD1 (G93A) mice	Spinal cord	↑	*Map2k3*, *Notch2* signaling pathway	[50,51,52]
miR-19a	SOD1 (G93A) mice	Brain	↑	*Rapgef2*	[24,31,50]
miR-19b	SOD1 (G93A) mice	Brain	↑		[31,50]
miR-21	SOD1 (G93A) mice/TDP-43 (Q331K) mice	Brain/Serum	Pathogenesis ↓	Inflammation-related	[27,47,53]
miR-29a	SOD1 (G93A) mice/ALS-FUS mice/TDP-43 (Q331K) mice	Spinal cord	↑	*Arpc3*	[31,54,55]
miR-29b family	SOD1 (G93A) mice/wobbler mice	Spinal cord/Brain	0 d ↓, 40 d ↑	*Bcl2* family	[56]
miR-30b-3p	FUS mice	Spinal cord/Hippocampus	↑		[55]
miR-17	SOD1 (G93A) mice	Spinal cord	↓	*Pten*, *Nedd4-2*, *Ndfip1*	[46]
miR-18a	SOD1 (G93A) mice	Spinal cord	↓		[46]
miR-20a	SOD1 (G93A) mice	Spinal cord	↓		[46]
miR-21a-5p	FUS mice/TDP-43 (Q331K) mice	Spinal cord/Serum	↑		[55]
miR-122-5p	TDP-43 (Q331K) mice	Hippocampus Cerebral cortex/Serum	↓	*Zfp827*, *Nfat5*, *Grhl2*	[31]
miR-124/a	SOD1 (G93A) mice	Spinal cord/Brainstem/Brain	Neural stem cell ↓, Differentiated neural stem cells ↑, brain ↑	*Sox2*, *Sox9*	[50,57]
miR-125b	SOD1 (G93A) mice	Spinal cord	23 w ↑	*A20*	[58]
miR-129-5p	SOD1 (G93A) mice	Spinal cord	15 w ↑	*USP10*/*Tp53*	[59,60]
miR-132	SOD1 (G93A) mice	Spinal cord	95 d ↓, 108 d ↓, 122 d ↓	*Bdnf*	[61]
miR-136-5p	TDP-43 (Q331K) mice	Hippocampus Cerebral cortex	↑		[31]
miR-138	SOD1 (G93A) mice	Spinal cord	Decreases daily starting at 126 d and maximizes at end stage	Not yet reported	[62]
miR-142-3p	SOD1 (G86R)	Serum	↑	*Tdp43*, *C9orf72*, *Cacna1d*, *Bcl2*	[47,63]
miR-146-5p	FUS mice	Spinal cord/Hippocampus	Spinal cord ↑ Hippocampus ↓	Inflammation-related	[55]
miR-146a	SOD1 (G93A) mice	Brain	Pre-onset ↓	Inflammation-related	[27]
miR-155	SOD1 (G93A) mice/C9ORF mice	Spinal cord/Brain	Asymptomatic stage ↑, Onset ↓	*Socs1*	[27,35,48]
miR-181	SOD1 (G93A) mice	Spinal cord	95 d, 108 d, 122 d ↑	*Caprin1*, inflammation-related, *Smad7*	[64]
miR-183-5p	TDP-43 (Q331K) mice/SOD1 (G93A)	Hippocampus Cerebral cortex/Serum	↑	Apoptosis and necrosis	[31,47]
miR-200c	SOD1 (G93A) mice	Spinal cord	15 w ↑	*Zeb1*	[59,65]
miR-200c-3p	TDP-43 (Q331K) mice	Hippocampus Cerebral cortex	↑		[31]
miR-204-5p	SOD1 (G86R)/SOD1 (G93A)/TDP-43 (A315T)	Serum	SOD1 (G93A) TDP-43 (A315T) ↓		[47]
miR-205-5p	SOD1 (G86R)/SOD1 (G93A)	Serum	SOD1 (G86R) ↓, SOD1 (G93A) ↑	*Zeb1*, *Zeb2*	[47]
miR-206	SOD1 (G93A) mice	Skeletal muscle	Decreased expression accelerates disease	*Pax7*, *Pax3*	[66,67,68]
miR-218	SOD1 (G93A) mice	Spinal cord	Decrease day by day from 126 d, maximum at end stage, no significant change in surviving neurons	Neuronal (MN) damage	[62]
miR-323-3p	FUS mice	Spinal cord/Hippocampus	↑	*Klf11*, *Ubap2*	[55]
miR-375-3p	SOD1 (G86R)	Serum	↓		[47]
miR-410-3p	FUS mice	Spinal cord/Hippocampus	↑	*Slc25a42*	[55]
miR-484	FUS mice	Spinal cord	↓		[55]
miR-486b-5p	TDP-43 (Q331K) mice	Hippocampus/Cerebral cortex/Serum	↑	*Zfp827*, *Nfat5*, *Grhl2*	[31]
miR-488-3p	FUS mice	Spinal cord/Hippocampus	↑	*Vapb*	[55]
miR-488-5p	FUS mice	Hippocampus	↑	*Vapb*	[55]
miR-496-3p	FUS mice	Spinal cord/Hippocampus	↑	*Mbnl1*, *Cyp26b1*	[55]
miR-1197-3p	FUS mice	Spinal cord/Hippocampus	↑	*Trib2*	[55]
miR-1249-3p	SOD1 (G93A)	Serum	↑	Axonal growth, ephrin B signaling pathway-related	[47]
